# Occurrence and multilocus genotyping of *Giardia intestinalis* assemblage C and D in farmed raccoon dogs, *Nyctereutes procyonoides*, in China

**DOI:** 10.1186/s13071-016-1771-3

**Published:** 2016-08-30

**Authors:** Xiao-Xuan Zhang, Wen-Bin Zheng, Jian-Gang Ma, Qiu-Xia Yao, Yang Zou, Cai-Jia Bubu, Quan Zhao, Xing-Quan Zhu

**Affiliations:** 1 State Key Laboratory of Veterinary Etiological Biology, Key Laboratory of Veterinary Parasitology of Gansu Province, Lanzhou Veterinary Research Institute, Chinese Academy of Agricultural Sciences, Lanzhou, Gansu Province 730046, People’s Republic of China; 2 College of Animal Science and Technology, Jilin Agricultural University, Changchun, Jilin Province 130118, People’s Republic of China; 3 Jiangsu Co-innovation Center for the Prevention and Control of Important Animal Infectious Diseases and Zoonoses, Yangzhou University College of Veterinary Medicine, Yangzhou, Jiangsu Province 225009, People’s Republic of China

**Keywords:** *Giardia intestinalis*, Genotyping, Prevalence, Raccoon dogs, China

## Abstract

**Background:**

*Giardia intestinalis*, the only causative agent of human giardiasis, can infect a wide range of animals. As no information concerning the prevalence and genotyping of *G. intestinalis* in raccoon dogs in China is available, examination of 305 faecal samples from raccoon dogs in Jilin Province (*n* = 110), Heilongjiang Province (*n* = 40), Liaoning Province (*n* = 72), Hebei Province (*n* = 54) and Shandong Province (*n* = 29) was conducted to estimate the prevalence of *G. intestinalis* in raccoon dogs in northern China and identify their genotypes using a genetic approach.

**Findings:**

Of 305 faecal samples from farmed raccoon dogs, 22 (7.21 %) were detected *G. intestinalis*-positive by nested PCR amplification of the triosephosphate isomerase (*tpi*) gene. The prevalence of *G. intestinalis* was strongly related to the region and season of sampling. All 22 samples were analysed at the *tpi*, the glutamate dehydrogenase (*gdh*) and the beta giardin (*bg*) gene loci, showing 13, 3, 2 subtypes, respectively. The results also demonstrated that two raccoon dogs harboured mixed infections of assemblage C and assemblage D (or mixed C/D), whereas only assemblage C was detected in the remaining 20 samples. Moreover, five new multilocus genotypes, named as MLGs C1-C5, were observed in the assemblage C in the present study.

**Conclusions:**

This is the first report of *G. intestinalis* infection in raccoon dogs in China. DNA sequence analysis of the *tpi*, *gdh* and *bg* gene indicated that 13, 3, 2 subtypes were found at these loci, respectively. Furthermore, this is also the first report of five new multilocus genotypes (MLGs C1-C5) in farmed raccoon dogs, which provides baseline data for further studies of the distribution of *G. duodenalis* in different hosts.

## Background

*Giardia*, comprised of six known species, is a protozoan genus with veterinary and public health importance [1–4]. Among these *Giardia* spp., *Giardia intestinalis*, a cosmopolitan zoonotic parasite, is the only causative agent of human giardiasis [4, 5]. Hosts acquire giardiasis mainly through faecal-oral route, and show symptom of diarrhoea [6, 7]. Approximately 280 million people are diagnosed *Giardia*-infected per year worldwide [3, 8]. Moreover, large numbers of organisms have also been reported as hosts of *G. intestinalis*, including raccoon dogs [9].

Eight genotypes or assemblages (A to H) of *G. intestinalis* have recently been described worldwide [2, 10, 11]. Assemblages A and B are responsible for most of human infections and also infect a wide range of non-human hosts [3, 12] and assemblages C–H seem to be animal-specific [3, 13, 14]. Generally, assemblages C and D were commonly found in dogs, but were occasionally identified in humans [2, 3, 13, 14].

China has rich diversity of animals, but limited information is available concerning the prevalence and genotypes of *G. intestinalis*. More importantly, no information is available for farmed raccoon dogs (*Nyctereutes procyonoides*) in China. The raccoon dog is an animal of economic importance for humans related to fur trading. In general, with the exception of raccoon dogs aged of less than 45 days (pre-weaned), raccoon dogs often feed in individual cages, consuming chicken’s intestines or fodder. The objectives of the present study were to reveal whether farmed raccoon dogs are infected with *G. intestinalis* in China, and to improve the information on the distribution of *G. intestinalis* assemblages in China.

## Methods

### Study population

The study population comprised of 305 raccoon dogs collected from 5 provinces in northern China, where nearly 25,100,000 raccoon dogs represented the breeding stock in 2015. According to the fact that prevalence of *Giardia* in dogs was 4.5 % in 2013–2014 [15], the expected prevalence is 4.5 % (P) with an accepted deviation of the true prevalence of 5 (*d*) and a confidence level of 95 % (*z* = 1.96). The sample size was calculated as 66 [according to *n* = P (1 − P)*z*^2^/*d*^2^].

### Specimen collection

A total of 305 faecal samples were randomly collected from farmed raccoon dogs in Jilin Province (*n* = 10), Liaoning Province (*n* = 72), Heilongjiang Province (*n* = 40), Hebei Province (*n* = 54), and Shandong Province (*n* = 29) in northern China in 2015. Samples were collected three times per year by seasons (three seasons from spring to autumn were defined as January-March, April-June, and July-September, respectively) from each of the eight farms, but probably not exactly from the same animals. Each of the samples was collected into a sterile disposal latex glove immediately after its defecation onto the bolster plates, and then transported to the laboratory. Information concerning region, season, gender and age were acquired.

### DNA extraction and PCR amplification

Genomic DNA was extracted from each of fecal samples using the Stool DNA kit (OMEGA, Norcross, Georgia, USA) according to the manufacturer’s instructions and stored at -20 °C until PCR test. Moreover, distilled water controls were included in each test to prevent/minimize cross-contamination at the DNA isolation or PCR phase. *G. intestinalis* prevalence and species/assemblages were detected by nested PCR amplification of approximately 530 bp fragment of the triosephosphate isomerase (*tpi*) gene. Furthermore, *tpi*-positive specimens were also analysed by PCR amplification of the glutamate dehydrogenase (*gdh*) and the beta giardin (*bg*) gene. The PCR amplification primers and their annealing temperatures for the three genes are listed in Table 1. Positive and negative controls were included in each test. Amplification products were observed under UV light after electrophoresis in 1.5 % agarose gel containing GoldView™ (Solarbio, Beijing, China).

**Table 1:** Primers used in the study, annealing temperatures used in the PCRs and expected sizes of the PCR products

Gene	Primer	Sequence (5′–3′)	Annealing temperature (°C)	Fragment length (bp)	Reference
*tpi*	F1	AAATIATGCCTGCTCGTCG	55	530	[3]
	R1	CAAACCTTITCCGCAAACC			
	F2	CCCTTCATCGGIGGTAACTT	55		
	R2	GTGGCCACCACICCCGTGCC			
*gdh*	F1	TTCCGTRTYCAGTACAACTC	50	530	[3]
	R1	ACCTCGTTCTGRGTGGCGCA			
	F2	ATGACYGAGCTYCAGAGGCACGT	65		
	R2	GTGGCGCARGGCATGATGCA			
*bg*	F1	AAGCCCGACGACCTCACCCGCAGTGC	50	511	[3]
	R1	GAGGCCGCCCTGGATCTTCGAGACGAC			
	F2	GAACGAACGAGATCGAGGTCCG	60		
	R2	CTCGACGAGCTTCGTGTT			

### Sequence and phylogenetic analyses

Positive secondary PCR products were sequenced by Sangon Biotech Company (Shanghai, China). All products were sequenced bidirectionally to confirm the accuracy of sequence. Meanwhile, genotypes that produced sequences with mutations, including single nucleotide substitutions, deletions or insertions, were confirmed by DNA sequencing of at least two PCR products. Assemblages and subtypes were identified by alignment of the nucleotide sequences with known reference *tpi*, *gdh* and *bg* gene sequences of *G. intestinalis* available in the GenBank database using BLAST (http://www.ncbi.nlm.nih.gov/BLAST/) and computer program Clustal X 1.83.

### Statistical analyses

The variation in *G. intestinalis* prevalence (*у*) of farmed raccoon dogs in relation to region, age, gender and season were analysed by *χ*^2^ test using SAS version 9.1 (SAS Institute Inc., USA). Results were considered statistically significant at *P* < 0.05. Odds ratios (ORs) and their 95 % confidence intervals (95 % CIs) were also calculated.

## Results and discussion

Of 305 raccoon dogs, 7.21 % (22/305) were tested *G. intestinalis*-positive in the present study, with 6.00 % in females and 8.39 % in males (Table 2). Statistically significant differences were found between autumn (3.51 %), spring (4.17 %) and summer (13.64 %) (*χ*^2^ = 10.62, *df* = 2, *P* = 0.0049) (Table 2). Raccoon dogs aged over 3 months (7.11 %) had similar prevalence than those of less than 3 months of age (7.50 %) (Table 2). Moreover, *G. intestinalis* prevalence in raccoon dogs of different region groups varied from 0 % to 15.28 %; the difference was statistically significant (*χ*^2^ = 11.69, *df* = 4, *P* = 0.0198) (Table 2). Prevalence in different farm groups ranged from 0 % to 16.67 % (Table 3). In the present study, the overall prevalence of *G. intestinalis* in farmed raccoon dogs was 7.21 % (22/305, 95 % CI: 4.31–10.12), a value much higher than that found in foxes in Croatia (4.5 %) [16] and Norway (4.8 %) [17]. The prevalence is also higher than that reported in a range of other animals in northern China, such as 3.63 % in dairy cattle in northwest China [3], 6.0 % in yaks in the central western region of China [18], 0.6 % in non-human primates in Henan Province [19], 5.0 % in sheep and goats in Heilongjiang Province [20], but slightly lower than that in golden takins (8.9 %) in Shannxi Province [21], police and farm dogs (13.2 %) in Shenyang, Liaoning Province [22] and rabbits (7.41 %) in Heilongjiang Province [23]. The difference may be related to many factors, such as different timing of specimen collection, different susceptibility to this disease, sample sizes, as well as different detection methods and climate at the sampling locations, so the real reason regarding this difference is difficult to explain.

**Table 2:** Prevalence of *Giardia intestinalis* in raccoon dogs in Jilin, Liaoning, Heilongjiang, Shandong and Hebei Provinces, northern China

Factor	Category	Number of positive/tested/	Prevalence (%) (95 % CI)	*P*-value	OR (95 % CI)
Region	Hebei Province	1/54	1.85 (0.00–5.45)	0.0198	Reference
	Heilongjiang Province	3/40	7.50 (0.00–15.66)		4.30 (0.43–42.94)
	Jilin Province	7/110	6.36 (1.80–10.93)		3.60 (0.43–30.05)
	Liaoning Province	11/72	15.28 (6.97–23.59)		9.56 (1.19–76.50)
	Shandong Province	0/29	0 (−)		–
Gender	Female	9/150	6.00 (2.20–9.80)	0.4205	Reference
	Male	13/155	8.39 (4.02–12.75)		1.43 (0.59–3.46)
Age	> 3 months	16/225	7.11 (3.75–10.47)	0.9081	Reference
	≤ 3 months	6/80	7.50 (1.73–13.27)		1.06 (0.40–2.81)
Season	Autumn	6/171	3.51 (0.75–6.27)	0.0049	Reference
	Spring	1/24	4.17 (0.00–12.16)		1.20 (0.14–10.38)
	Summer	15/110	13.64 (7.22–20.05)		4.34 (1.63–11.57)
Total		22/305	7.21 (4.31–10.12)

**Table 3:** Distribution of *Giardia intestinalis* genotypes in different farms

Region	Farm ID	Sample size	Prevalence (%)	Genotype ID (no.)
Jilin Province	1	80	7.50	C1 (1); C2 (4); C9 (1)
	2	30	3.33	C10 (1)
Hebei Province	3	30	0	0
	4	24	4.17	C12 (1)
Liaoning Province	5	42	16.67	C4 (2); C6 (2); C7 (1); C11 (1); C13 (1)
	6	30	13.33	C8 (1); C11 (3)
Shandong Province	7	29	0	0
Heilongjiang Province	8	40	7.50	C3 (1); C5 (2)
Total		305	7.21	C1 (1); C2 (4); C3 (1); C4 (2); C5 (2); C6 (2); C7 (1); C8 (1); C9 (1); C10 (1); C11 (4); C12 (1); C13 (1)

Faecal-oral route is the most important way of *G. intestinalis* transmission [6]. Therefore, higher raccoon dog density in Liaoning Province is one of the most important reasons why raccoon dogs from Liaoning have a higher *G. intestinalis* prevalence than those from other provinces (*P* = 0.0198) (Table 2). A previous study suggested that higher precipitation can create more opportunities for *G. intestinalis* transmission [3]. This is supported by the higher *G. intestinalis* prevalence detected in summer and spring in the present study (Table 2).

A total of five *G. intestinalis* assemblages, namely assemblages A, B, C, D and E, have been found in canids worldwide [24–26], and in some cases these were detected as mixed infections. In order to estimate the real state of *G. intestinalis* infections and determine whether mixed infections exist in the raccoon dogs examined here, we applied multilocus genotyping (*tpi*, *gdh* and *bg* loci). However, probably due to the smaller sample size, only assemblages C and D were found based on three loci and 22 *tpi*, 13 *bg* and 11 *gdh* gene sequences were acquired. These results suggest that the raccoon dog population studied exhibits a lower risk for transmission of zoonotic *G. intestinalis* to humans in the investigation sites. The fact that only two raccoon dogs were identified with mixed infections (assemblages C and D) whereas the remaining samples were infected only with assemblage C further confirm that C and D are the most prevalent *G. intestinalis* assemblages in canids.

In this study, all 22 isolates of *G. intestinalis* were characterized at the *gdh*, *tpi* and *bg* gene loci, and high genetic polymorphism of *G. intestinalis* was observed at the three loci (Table 4). Among 13 *tpi* subtypes, with the exception of the sub-assemblage C4 (accession no. KX014798) previously reported from a pig in China (accession no. KJ668133), each of the remaining 12 sub-assemblage sequences (C1–C3 and C5–C13) had 99 % similarity with the reference sequence of assemblage C, which have not been recorded previously. Moreover, six and ten SNPs were observed at *gdh* and *bg* loci, respectively. At *gdh* locus, three sub-assemblages (C1–C3) were detected, with two known sub-assemblages (C1 and C3) and one novel sub-assemblage (C3). Two (C1, KX014790 and C2, KX014791) of them were identical to the known sequences of assemblage C: accession no. JN587353 (from dog in Croatia [27]) and accession no. KF993732 (from Husky dog in China [28]). Assemblage C3 (accession no. KX014792) has not been reported previously, and the sequence showed 99 % similarity with the reference sequence (accession no. KF993732, from dog in China [28]). Furthermore, two assemblages (C and D) were identified at *bg* locus, with one sub-assemblage C1 and one sub-assemblage D1. The two sub-assemblages, C1 (KX014788) and D1 (KX014789), were reported in *Canis lupus familiaris* in Belgium previously [29]; our sequences exhibited 100 % similarity with the reference sequences of assemblage C (accession no. HM061150, from *Canis lupus familiaris* in Belgium [29]), and assemblage D (accession no. HM061152, from *Canis lupus familiaris* in Belgium [29]), respectively. Six *G. intestinalis* isolates were successfully sequenced at all three loci, forming five new multilocus genotypes (MLGs) in assemblage C, namely MLGs C1–C5 (Table 5). All five different assemblage C MLGs were identified for the first time, which may be due to the differences in species susceptibility and geographical locations. These findings can provide baseline data for further studies of the analyzation of *G. duodenalis* assemblage MLGs. These results also indicate high genetic diversity of *G. intestinalis* assemblage C in raccoon dogs in China, which agree with previous reports showing that the same *G. intestinalis* assemblage isolates may be divided into different MLGs [3, 21].

**Table 4:** Variations in the *tpi*, *gdh* and *bg* nucleotide sequences among the genotypes of the *Giardia intestinalis* in raccoon dogs in Northern China

Locus	Subtype (no.)	Nucleotide at position												GenBank accession no.
*tpi*		3	9	10	99	163	189	317	368	378	423	444	521	
	18–2 (1)	C	T	C	C	C	C	T	T	A	G	G	G	KX014795
	WH10 (4)	T	T	C	C	C	C	T	T	C	G	G	G	KX014804
	Z27 (1)	T	T	G	C	C	C	T	T	C	G	T	G	KX014803
	A38 (2)	C	C	G	T	C	C	T	C	A	A	G	T	KX014798
	Z31 (2)	C	C	G	C	C	C	T	C	A	A	G	T	KX014799
	A6 (2)	C	C	G	T	C	C	T	C	A	A	G	G	KX014797
	A22 (1)	C	T	C	T	C	C	T	T	A	A	G	G	KX014796
	L24 (1)	C	C	G	T	C	C	T	T	A	G	G	G	KX014800
	11–1 (1)	C	C	G	C	C	C	C	T	A	G	T	G	KX014801
	L21 (1)	C	C	G	C	C	C	C	T	A	A	G	G	KX014802
	L15 (4)	C	C	G	C	T	T	C	T	C	G	G	T	KX014793
	L10 (1)	G	G	G	C	T	T	C	T	C	G	G	G	KX014794
	A11 (1)	C	C	G	C	T	T	C	T	A	G	G	G	KX014805
*gdh*		22	112	115	126	151	494							
	18–2 (6)	C	C	A	T	G	C							KX014790
	11–1 (4)	A	C	A	T	A	C							KX014791
	A14 (1)	C	T	G	C	G	A							KX014792
*bg*		18	27	45	54	60	72	78	96	97	102			
	11–1 (11)	C	C	C	T	G	C	C	G	G	G			KX014788
	Z27 (2)	T	T	T	A	T	T	G	A	A	C			KX014789

**Table 5:** Multilocus characterisation of *Giardia intestinalis* assemblage C isolates from raccoon dogs at *tpi*, *gdh* and *bg* loci

Isolate (no.)	Genotype	GenBank acc. no.	MLG
18–2 (1)	C1, C1, C1	KX014795, KX014790, KX014788	MLGC1
A14 (1)	C4, C3, C1	KX014798, KX014792, KX014788	MLGC2
Z31 (1)	C5, C2, C1	KX014799, KX014791, KX014788	MLGC3
11–1 (1)	C9, C2, C1	KX014801, KX014791, KX014788	MLGC4
L15 (2)	C11, C1, C1	KX014793, KX014790, KX014788	MLGC5

## Conclusions

The present study demonstrated the existence (7.21 %, 22/305) of *G. intestinalis* in farmed raccoon dogs in China. This study also found *G. intestinalis* assemblages C and D in farmed raccoon dogs by MLG model for the first time, with 13, three and one genotypes of sub-assemblage C at the *tpi gdh* and *bg* loci, respectively. Moreover, five new MLGs (MLGs C1–C5) were found in the present study. These findings have implications for further studies of the distribution of *G. intestinalis* in different hosts.

## Abbreviations

*bg*: Beta giardin gene
CI: Confidence interval
*gdh*: Glutamate dehydrogenase gene
MLG: Multilocus genotype
OR: Odds ratio
*tpi*: Triosephosphate isomerase (*tpi*) gene
